# A Reply to Some of the Arguments That Have Been Advanced to Overthrow the Comma Bacillus of Asiatic Cholera*Read before the American Association of Microscopists, at Chautauqua, August, 1886.

**Published:** 1886-10

**Authors:** George W. Lewis


### *A REPLY TO SOME OF THE ARGUMENTS THAT HAVE BEEN ADVANCED TO OVERTHROW THE COMMA BACILLUS OF ASIATIC CHOLERA.

By GEORGE W. LEWIS, Jr., A. M., M. D.

On the 25th of July, 1884, Dr. Robert Koch addressed the Imperial German Board of Health, at Berlin, on the subject of cholera and its bacillus. He there expressed his belief that the comma bacillus bears the relation to the cholera process of cause and effect; that the organism gains entrance by the mouth, and depends, for a safe passage through the stomach, upon some disordered condition of that organ or its function; that the real seat of the disease is the lower part of the ileum, including Peyer's glands; and that, up to that time, inoculation experiments had given only negative results.

He pointed out, moreover, a striking relationship between the course of the disease and the life-history of the organism, both as regards the remarkably short time required for it to reach a full development and the equally rapid decline which immediately sets in. His statements were the more valuable because they were based upon some three hundred autopsies, performed in the height of cholera epidemics in France, Egypt and India, and because, not satisfied even with this, he had visited the long stretch of country known as the Delta of the Ganges, where the disease prevails, to a greater or less extent, throughout the entire year.

From the date of that memorable meeting to the present time, the comma bacillus has been a favored target at the hands alike of the skilled and unskilled in this branch of medical research. To attempt an answer to some of the so-called arguments that have been advanced to overthrow the comma bacillus and its relation to the cholera process would be a mere waste of time, for the promulgators of these theories show, by their methods of reasoning, their utter ignorance of even the

*Read before the American Association of Microscopists, at Chautauqua, August, 1886.

principles upon which the science of bacteriology is based. Such, for example, are those who claim a spontaneous origin for the disease, and likewise those who assert, apparently without any foundation, that the comma bacillus is only a transition stage in the development of the organism which really causes the pathogenic changes. This idea, I believe, grew out of the fact that inoculation experiments were not altogether positive in their results, and, although it is generally conceded that the comma bacillus is, in some way, inseparably associated with the disease, those who dissent from the idea of its being the *causa causans* prefer to class the comma bacillus itself as the harmless stage in the life-history of the organism, which, at an earlier or later stage, constitutes the *materies morbi*. So far as I know, there is no instance on record of the transformation of one kind of bacteria into another, and in so brief an existence as that of the comma bacillus, where the stages of its life-history must necessarily follow each other in rapid succession there could not be one pathogenic stage while all the rest are harmless. It is true that some bacteria, for example anthrax bacilli, lose their pathogenic properties when subjected to the influence of certain re-agents, but a change of form has never been noticed. The above, however, is an illustration diametrically opposed in character to the one of which we are speaking. Here we would have a harmless intestinal bacterium take on, instead of throw off its pathogenic characteristics. Another point not to be overlooked in this connection is that the proportion of comma bacilli to other intestinal bacteria has, in some cases, been rated as high as ten to one, which would imply that during its brief stage of harmlessness its power to multiply is increased ten fold. This, of course, is contrary to any known law.

Another theory is, that the comma bacillus is the scavenger rather than the cause of the disease; that is, conditions are created by the disease through which, among the many bacteria that are to be found in the intestine, one kind or another is changed, and assumes the qualities observed in the comma bacillus. The explanation given in the above case is equally applicable here. Our knowledge of bacteria shows us that, so far as form is concerned, they are all remarkably constant. If they were not, successive cultivations outside of the body would cause them, sooner or later, to revert to their original form. The comma bacillus has been carried as high as the twentieth cultivation in various nutrient media, and yet no alteration, either in form or manner of growth, has been observed. If it were only a transitional stage of some bacterium, that fact would certainly be revealed during successive cultivations. Likewise, if it is the scavenger of the cholera process, then, as soon as it is separated from those peculiar surroundings, the germs would cease to flourish. But no, it is constant throughout.

It is Klein, I believe, who expresses the view that the cholera process favors the growth of comma bacilli by preparing a nutritive soil for them, and, on this hypothesis, he endeavors to explain the striking increase of this particular kind of bacteria. If this is so, then we must start with the understanding that the comma bacillus exists in the perfectly healthy body; but the most searching investigations have failed to reveal the slightest trace of an organism that in any way resembles it, either in form or manner of growth. Moreover, in cases which eventually terminated in recovery, there was manifestly a decrease in the number of comma bacilli corresponding to the stage of convalescence.

Of course, if it were possible to induce the choleraic condition in animals by the inoculation of comma bacilli, there would be no room for controversy as to the causative power of this organism. Even if, by feeding appreciable quantities of this form of bacteria, any derangement of the digestive process was brought about, it would at least give encouragement as to its causative properties. So far from having either of these effects, its action is not perceptible. This seems a strong barrier, and, were it possible to bring to account the case of a single animal fallen ill with the disease during any of the epidemics, it would certainly be an insurmountable obstacle to the consideration of the causative relation of this organism. But, from all the quarters that have been visited by epidemics, come unanimous reports as to the exemption of animals from a condition in any way resembling cholera. This carries with it a good deal of weight which alleviates, in a degree, the disappointment resulting from the failure of inoculation experiments. It was evident, from careful examination of the intestines, that the comma bacilli had been killed during their passage through the stomach; in fact, few were found in the intestinal canal, being confined, for the most part, to the walls of the stomach. It is probably true, too, that if the digestive tract of man is free from derangement, he will likewise be able to resist the disease. When Klein, of England, swallowed a considerable quantity of a pure culture of comma bacilli, and thought by so doing that he would prove or disprove the claims made for this organism, he really proved nothing positive, for this reason: Pre-disposition has always played a most important part in cholera infection. Statistics show that by far the largest proportion of cases occur on Mondays and Tuesdays; that is, on days which have been preceded by excesses in eating and drinking.

While under Koch's tuition, at Berlin, in the winter of 1884, the writer busied himself particularly in the line of inoculation experiments, being quite as skeptical as any one could be in regard to the claims made for the comma bacillus. The animals used for this purpose were white mice, guinea pigs and rabbits. No effect whatever was produced by feeding a pure culture, but when the bacilli were introduced directly into the duodenum, the guinea pigs and rabbits, some six hours later, manifested most of the symptoms of cholera infection. The mice, however, exhibited no trustworthy symptoms, dying apparently from indisposition. Upon examination of the intestine, after death, large numbers of comma bacilli were found in the region of Peyer's glands, the glands themselves being enlarged and inflamed. These appearances were very distinct in the guinea pigs and rabbits, but scarcely perceptible in the mice. From a physiological standpoint, there is no apparent reason why the mice should be effected differently from the guinea pigs and rabbits, and yet, throughout Germany, experiments upon mice have been unsatisfactory. The small size of the animal, and the consequent care that must be taken in reaching a desirable point of inoculation will explain in part, I think, the failure in a large number of cases. This is evident because a great many of the mice die from peritonitis rather than from any direct effect exerted by the bacilli.

If we assume that a disease is caused by a specific germ, we cannot think of an autochthonous origin emanating from any particular locality. Such a specific organism must follow the laws of vegetation just as the most highly developed plant. It must always propagate itself from something of the same nature, and cannot spring up at hap-hazard from other things, or from nothing. In the case of the comma bacillus, whose home has been quite closely defined, we are forced to trace back the disease that depends upon it to special localities from which this specific micro-organism is brought to us.

